# Biventricular assist devices and total artificial heart: Strategies and outcomes

**DOI:** 10.3389/fcvm.2022.972132

**Published:** 2023-01-06

**Authors:** Taiyo Kuroda, Chihiro Miyagi, Kiyotaka Fukamachi, Jamshid H. Karimov

**Affiliations:** ^1^Department of Biomedical Engineering, Lerner Research Institute, Cleveland Clinic, Cleveland, OH, United States; ^2^Cleveland Clinic Lerner College of Medicine of Case Western Reserve University, Cleveland, OH, United States

**Keywords:** biventricular heart failure, left ventricular assist device, heart transplantation, Impella^®^, right heart failure, HeartMate 3^®^, Syncardia^®^ TAH, ProtekDuo^®^

## Abstract

In contrast to the advanced development of the left ventricular assist device (LVAD) therapy for advanced heart failure, the mechanical circulatory support (MCS) with biventricular assist device (BVAD) and total artificial heart (TAH) options remain challenging. The treatment strategy of BVAD and TAH therapy largely depends on the support duration. For example, an extracorporeal centrifugal pump, typically referred to as a temporary surgical extracorporeal right ventricular assist device, is implanted for the short term with acute right ventricular failure following LVAD implantation. Meanwhile, off-label use of a durable implantable LVAD is a strategy for long-term right ventricular support. Hence, this review focuses on the current treatment strategies and clinical outcomes based on each ventricle support duration. In addition, the issue of heart failure post-heart transplantation (post-HT) is explored. We will discuss MCS therapy options for post-HT recipients.

## 1. Introduction

The clinical data of mechanical circulatory support (MCS) for biventricular heart failure (BHF) is limited due to the low prevalence of treating severe right heart failure (RHF). The complexity of the BHF treatment strategy also makes the situation challenging. The reason for the complexity is that the BHF treatment strategy will be determined with the consideration of support duration for both ventricles. Furthermore, the clinical scenario will be important to determine the device; for example, the device selection may differ with the Interagency Registry for Mechanically Assisted Circulatory Support (INTERMACS) patient profiles and baseline patient characteristics, including the diagnosis (1, 2).

BHF support conditions have combinations of chronic/temporary support duration and left/right ventricle, which is as follows: (1) Chronic left ventricular (LV) support with chronic right ventricular (RV) support; (2) Chronic LV support with temporary RV support; (3) Temporary LV support with chronic RV support; and (4) Temporary LV support with temporary RV support. In this review, we will focus on 1, 2, and 4, because the temporary LV support with chronic RV support situation is quite rare due to RHF pathogenesis (3).

Lastly, this review article will discuss populations in need of BHF support among post-heart transplantation (post-HT) patients (4). Since the first successful human heart transplantation (HT) reported in 1967 in South Africa, over 120,000 patients have received HT therapy (5). HT volumes have been increasing steadily for more than a decade (6). Also, the survival rate is improving, as median survival after adult HT performed in 2002–2008 now exceeds 12 years; therefore, the number of post-HT patients are increasing. However, the leading causes of death among post-HT patients, remain the rate of graft failure and infection (6). We will discuss the possibility of indications for BHF support MCS in this situation.

## 2. Devices for chronic LV support with chronic RV support

There are two options for chronic LV support with chronic RV support: using a continuous-flow (CF) left ventricular assist device (LVAD) and using one more LVAD as a right ventricular assist device (RVAD); or a total artificial heart (TAH). Patients requiring RVAD after LVAD implantation, or lower INTERMACS patient profile patients with biventricular failure diagnosis, are the most frequent example for this clinical situation.

Regarding the first listed population (patients requiring RVAD after LVAD implantation; biventricular assist device, BVAD), according to the Twelfth INTERMACS Report, approximately 2,700–3,000 patients in the United States (US) receive an LVAD implant each year; and, in 2020, CF LVADs have accounted for the most (7). In the HeartMate 3 ^®^ (HM3) (Abbott, Abbott Park, IL, USA) pivotal and post-pivotal trial study, 4.1 and 7.4% of patients, respectively required BVAD (8). Several reasons for RHF during the LVAD therapy have been hypothesized. For instance, the shift of the interventricular septum (IVS) to the left side by LVAD therapy reduces the septal contribution of the RV contraction and thus increased workload is a concern for worsening RV function (9–12). In addition, the shift of the IVS may distract the septal papillary muscle with systolic restriction of septal leaflet motion, which may intensify the tricuspid regurgitation (13). Furthermore, increased venous return created by increased cardiac output from the LVAD may worsen the potential RV dysfunction that LVAD patients already have to some degree (14).

One of the keys to a successful BVAD therapy may be estimating the support duration. In HM3 patients, approximately 40% of those upgrading to BVAD were performed within 0–2 days after HM3 implantation, and 23% of upgrades were performed within 3–14 days (8). The severe late RV failure among LVAD patients, which is defined by the requirement for an RVAD at 3–12 months from LVAD implant, is very rare (15). In addition, over 60% of successful RVAD weaning rates were reported with a median support duration of 13–17 days (16–19). Therefore, paradoxically, if BVAD intervention after LVAD implantation took place in an earlier period, BVAD support duration is expected to be short (up to 17 days). An investigation using the United Network for Organ Sharing (UNOS) database showed that 1% of LVAD patients were transitioned to durable BVAD support, and 0.2% of LVAD patients were transitioned to TAH support (20). Appropriate timing for the intervention is also critical. Preoperative hemodynamic parameters are used to assess the RV function after LVAD implantation (Table 1) (21). For example, with preoperative central venous pressure (CVP) greater than 15 mm Hg and CVP/pulmonary capillary wedge pressure (PCWP) greater than 0.63, a significantly higher risk of RHF was reported (17). The preoperative RV stroke work index (RV SWI) was reported as another predictor for RVAD implant function, which suggests that RV SWI was lower in the RVAD group (22, 23). The preoperative pulmonary artery pulsatility index was reported as a predictor for early RV failure with a cutoff value of 2.0 (23, 24). Also, using echocardiography, the RV global longitudinal strain predicted an early acute and post-implant RV failure with a cutoff value of −9.7% (25). However, a single parameter still may not be sufficient in predicting the RHF after LVAD implantation (26).

**TABLE 1:** Preoperative predictors for right ventricle failure following left ventricular assist device (LVAD) implantation.

Parameters	Description
CVP > 15 mm Hg	OR: 2.1 (IQR 1.2–3.6)
CVP/PCWP > 0.63	OR: 2.5 (IQR 1.4–4.6)
RV SWI	RVAD [151 ± 75 (mm Hg × mL/m^2^)] > no RVAD [368 ± 245 (mm Hg × mL/m^2^)] (*p* = 0.01)
PAPi < 2.0	AUC, 0.77; sensitivity, 74%; specificity, 67%
RV GLS > −9.7%	AUC, 0.86; sensitivity, 89%; specificity, 78%

CVP, central venous pressure; OR, odds ratio; IQR, interquartile range; PCWP, pulmonary capillary wedge pressure; RV SWI, right ventricle stroke work index; RVAD, right ventricle assist device; PAPi, pulmonary artery pulsatility index; AUC, area under curve; RV GLS, right ventricle longitudinal strain.

Using durable implantable CF LVAD as RVAD, such as HM3, currently is an off-label use. There is a lack of a proven, long-term MCS devices except for the dual Berlin Heart EXCOR ^®^ system (Berlin Heart, GmbH, Berlin, Germany), which is mainly used in pediatric patients. Due to anatomical and physiological limitations (12), a modified technique was used to implant dual HM3 (27, 28). The clinical outcome for dual HM3 varied among two studies; the survival at 18 months was 54.6–91.7% (28, 29). Another retrospective study using INTERMACS database (*n* = 38, multi-center) described that survival outcomes among the BVAD patients (BVAD was defined as LVAD and RVAD implanted in the same operation) were 68% at 6 months and 62% at 12 months (30). This study found that 11 patients died with device in place, 9 patients survived to reach HT, and 18 patients were alive on support at the mean follow-up of 5.08 months.

**FIGURE 1:**
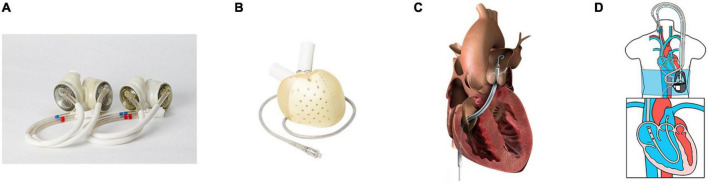
Mechanical circulatory support (MCS) devices for right ventricular/biventricular failure. **(A)** SynCardia^®^ total artificial heart 70 cc (left) and 50 cc (right), re-use from Villa et al. (60), **(B)** Aeson^®^ total artificial heart (Image from Carmat SA, used with permission), **(C)** Impella RP^®^ (Image from Impella RP instruction manual, used with permission), and **(D)** LifeSparc^®^ pump with ProtekDuo^®^ cannula (Image from Livanova Investor Day 2021 presentation, used with permission).

Another option for chronic LV support with chronic RV support is TAH. SynCardia^®^ TAH (Figure 1A) (SynCardia Systems. LLC, Tucson, Arizona, USA) is the most commonly used TAH. It is a pneumatically driven FDA-approved, volume displacement pump TAH with a size line-up of 50 mL and 70 mL. The clinical data reported that the TAH patient mortality while waiting for HT was 7.4%, while 87% of patients reached HT (20). An analysis from the INTERMACS database (*n* = 450, all patients received SynCardia 70 ml TAH) showed that survival at 1 year was 53.2% and 33.9% at 2 years (2). HT was performed in 266 patients, and 162 patients died on support. Among those, 80% of patients in this study were INTERMACS patient profiles 1 or 2, and 20% of patients were supported with extracorporeal membrane oxygenation (ECMO). Notably, survival was superior in the earlier era (2006–2011) than the most recent era (2012–2017), which suggests that the difference in survival was largely influenced by the experience of the facility (31); therefore, the TAH survival may improve with surgical and patient management experience including the patient selection and the timing of intervention (2). Furthermore, the baseline condition of the TAH cohort was sicker than LVADs patients, which may have affected the survival. Currently, the largest difference between BVAD and TAH is the strategy implemented. TAH implantation procedure resect both ventricles; therefore, a bridge to recover treatment concept is not feasible, and HT is the only way forward. However, destination therapy with SynCardia 70 mL TAH, in which a clinical trial is ongoing in 2022, may change the future strategy.

Aeson^®^ TAH (Figure 1B) (Carmat SA, Vélizy-Villacoublay, France), is a volume displacement, electro-hydraulically driven TAH which has recently been approved by the FDA to conduct an early feasibility study (32). Currently, the only clinical data available for Aeson^®^ TAH is an implantation report for four patients, which describe a support period of 20–270 days, with two patients being discharged to return home (33). In addition, several TAHs are in development, such as Cleveland Clinic CF TAH (CFTAH) (Cleveland Clinic, Cleveland, OH, USA) (34–36), BiVACOR^®^ TAH (BiVACOR, Inc., Houston, TX, USA) (37–39), and Realheart^®^ TAH (Scandinavian Real Heart AB, Vesteras, Sweden) (40–42).

## 3. Devices for chronic LV support with temporary RV support

For chronic LV support with short to intermediate RV support duration (0–30 days), an extracorporeal centrifugal pump has been widely used. Customarily, this configuration is called a surgical extracorporeal RVAD (sRVAD). In sRVAD, the inflow cannula is placed directly, or (*via* the femoral vein) into the right atrium (RA), and the outflow cannula is placed directly, or (*via* a sutured prosthesis graft) into the pulmonary artery (PA) (16, 43). Both cannula will pass through the skin, and the circuit will be connected to a centrifugal pump system, such as the CentriMag^®^ blood pump (Abbott, Abbott Park, IL, USA). The advantage of this system is that it is easy to place, so if RV failure presents during the HM3 implantation procedure, the sRVAD can be placed through a sternotomy. Also, this system is able to provide much larger flows than a veno-arterial extracorporeal membrane oxygenation system. The disadvantage of this system is that it may require a re-sternotomy to explant the sRVAD, which increases the risk of infection.

The RVAD implantation following LVAD implantation occurs within 0–2 days (8), and a study reported that 86% of patients who received sRVAD after LVAD implantation were successfully weaned from support with a duration of 16.4 ± 9.6 days (44). Another analysis using the INTERMACS database reported that the 1-, 6-, and 12-month survival rates for the chronic BVAD patients were 89, 68, and 62%, respectively, and there was no significant difference between the patients with chronic LV support with temporary RV support (30).

Sternotomy for HM3 implantation allows easy access to both RA and PA for sRVAD implantation; however, an effort to implant HM3 with minimally invasive surgery, preferably by thoracotomy, is under review (45); therefore, access to the RA and PA would be limited. Since the most common support duration is temporary to intermediate (0–15 days), percutaneous RV assist device (p-RVAD), such as Impella RP^®^ (Figure 1C) (Abiomed, Danvers, MA, USA) and LifeSPARC^®^ Pump with ProtekDuo^®^ dual-lumen cannula (Figure 1D) (LivaNova, Houston, TX, USA), may be a good solution. A prospective cohort study, which includes patients who received Impella RP implantation following LVAD implantation (*n* = 31), described that 77.4% of patients reached the primary end-point which was survival at 30 days, or discharge after device explant, or induction of anesthesia for a long-term therapy (46). Another retrospective study reported that 27 LVAD patients received the LifeSPARC Pump with ProtekDuo dual-lumen cannula system implantation, and device weaning occurred in 86% of patients, with 15% resulting in-hospital mortality (47). Those outcomes seems acceptable; thus, in cases of unplanned p-RVAD implantation, and if high-risk RV failure patients are having surgery, it may be better to perform LVAD implantation surgery in the hybrid operating room.

## 4. Devices for temporary LV support with temporary RV support

There are emergent, acute scenarios that require temporary BVAD support. The percutaneous LVAD (p-LVAD), such as Impella^®^ (Abiomed, Danvers, MA, USA), has been increasingly used as temporary LV support, and sometimes it is used in combination with a p-RVAD. In a retrospective study among 5 U.S. hospitals (*n* = 20),with a combination of left side Impella and Impella RP, called BiPella (48), it was reported that in-hospital mortality was 50% (49). The advantage of this system is its ease to implant and explant the device; however, a concern of this therapy is adequate LV unloading and it should be monitored to ensure appropriate support is supplied. If not, the pulmonary vasculature is over-pressurized due to the high pulmonary resistance resulting from high left atrial pressure. The combination of left side Impella and LifeSPARC Pump with ProtekDuo dual-lumen cannula has been described in several reports (21, 50–52).

## 5. Devices for post-heart transplantation biventricular failure

HT is one of the *de facto* goals of current end-stage HF treatment, which has an overall median survival of 12.5 years, and a conditional survival curve of 14.8 years for those who survived the first year (53). Hospitalization due to late graft failure was observed in 33% of patients (54). Among those populations, some may progress to cardiogenic shock, and may need a MCS. Notably, a retrospective study (*n* = 26) reported that 42% of patients with late graft failure were treated with BVAD and TAH (4). Among the patients with BVAD implantation in this study, 60% received dual CentriMag configuration, 20% received dual implantable LVAD configuration (details unknown), 10% received dual HVAD, and 10% received HVAD with sRVAD. The outcomes were that 23% were weaned (including single VA-ECMO), 19% underwent Re-heart transplantation (Re-HT), and 15% were discharged with durable MCS. The mortality rate was 42%.

Re-HT may be an option for patients who develop refractory graft failure. The indication is rare, but the population receiving second and third Re-HT has increased (55). Although MCS is not commonly performed as a bridge to Re-HT, the TAH is theoretically an ideal option because the antigen would be removed from the body; however, the outcome for the TAH as a bridge to Re-HT is reported as very high risk, and the potential for improving survival remains uncertain (56). In addition, TAH as a destination therapy may change future treatment strategies.

## 6. Discussion

In this review, we mainly discussed biventricular failure with post-LV failure pathogenesis based on the classification of support duration. The treatment strategy differed in support duration, and there is an interesting, ongoing effort in both acute and chronic biventricular MCS. In addition, congenital heart disease is still another condition that may require biventricular support. The most widely used MCS among congenital heart disease population is the Berlin Heart EXCOR^®^ system, which offers a variety of pump sizes. Treatment strategies for the pediatric population may differ due to patient growth.

Regarding chronic BVAD, dual HM3 is the most commonly used configuration. The adverse event most noted is RVAD pump thrombosis, which has been consistently reported with occurrence of 36–37% (12, 57, 58). However, it seems lesser than previous reports (28, 29), which can be explained by the low thrombosis risk of the HM3. HM3 is a centrifugal pump with a displacing volume of 80 mL, and it is slightly larger than HeartWare^®^ Ventricular Assist Device (HVAD) (Medtronic Inc., Minneapolis, MN, USA) (50 mL); this size difference influences device location inside the patient’s chest. HM3 is likely to be implanted into the free wall of the RA. The pump is wrapped in Gore-Tex^®^ (W.L. Gore, Flagstaff, AZ, USA), or a polytetrafluoroethylene sheet, and is placed as it protrudes into the right thoracic cavity through a slit in the pericardium (27, 29). Although the relationship with pump positioning was uncertain, the evacuation of a right hemothorax, effusion, and Aspergillus species infection in the RVAD cavity were reported. On the other hand, HVADs were implanted into either the RA (38%) or RV (63%) (59). RA-implanted HVAD was supported longer than RV HVAD (*p* = 0.02), and did not show significant differences in postoperative complications, such as pump thrombosis. Therefore, a small, durable pump implanted into the RA appears to be a viable option for RVAD used in chronic BVAD.

In conclusion, the percutaneous temporary RVAD may increase its prevalence in temporary biventricular support. As for long-term biventricular support, the development of a durable, specifically designed RVAD, with a wide operation range and suitable inflow cannulas, is expected. Furthermore, the development of a durable BVAD, including TAH, may provide a valid solution for the management of heart failure among post-HT patients.

## Author contributions

TK: manuscript preparation. CM: critical manuscript review. KF: critical revision of article. JK: critical revision and approval of article. All authors contributed to the article and approved the submitted version.
